# Intra-abdominal mass with empty scrotum in adult male revealed as testicular seminoma: A case report^☆^

**DOI:** 10.1016/j.radcr.2022.06.038

**Published:** 2022-07-11

**Authors:** Yahye Garad Mohamed, Najib Mohamed Salad, Abdinasir Mohamed Elmia, Abdihamid Mohamed Ali

**Affiliations:** a Radiology Department, Mogadishu Somali Turkey, Recep Tayyip Erdogan Training and Research Hospital, Mogadishu, Somalia; b General Surgery Department, Mogadishu Somali Turkey, Recep Tayyip Erdogan Training and Research Hospital, Mogadishu, Somalia

**Keywords:** Intra-abdominal, Testicular, Seminoma, A Case report

## Abstract

During fatal development, the testes grow in the abdomen and descend into the scrotal sac. It can be stopped at any point along its path (cryptorchidism) or migrate to an atypical side (ectopic testis).

A 47-year-old man from Somalia's lower Shabelle region was presented to the urology OPD, He had a history of non-tender abdominal mass for the previous four months, An examination revealed a mass in his abdomen that was firm, non-tender, and immobile, an abdominal ultrasound and a contrast enhanced CT abdomen showed a 15-cm heterogeneous bean-shaped mass above the bladder.

After patient counseling and informed written consent, a laparotomy was done to remove the tumor. A seminoma of the undescended testis was identified during a histological examination.

An intra-abdominal mass with empty scrotum should raise concerns about an intra-abdominal testicular tumor. To prevent/early discover these types of tumors, cryptorchidism should be treated at a young age, particularly before to the first year.

## Background

The testes grow in the abdomen throughout fatal development and then descend into the scrotal sac in the third trimester, usually between weeks 28 and 32. It may be stopped at any time along its path (cryptorchidism) or migrate to an abnormal side throughout the descent (ectopic testis) [1].

The absence of one or both testes from the scrotum is known as cryptorchidism. It is the most frequent male genital birth defect, with an incidence of 3% in full-term male new-borns compared to 30% in preterm male neonates making it one of the most frequent congenital abnormalities [2], [3], [4]. Between the ages of 6 months and 1 year, the incidence drops to 1% [2].

The cryptorchid testis predisposes to testicular cancer, ischemia, and infertility later in life. Testicular seminoma is the most prevalent malignant transformation of an undescended testis [2,5].

## Case presentation

A 47-year-old man from Somalia's lower Shabelle region presented to the urology Outpatient Department with a history of a non-tender abdominal mass for the previous 4 months, gradually increasing in size for 3 months and rapidly growing for the last 10 days. He also described a history of sexual dysfunction, libido loss, and infertility. There was no history of pain radiation, no aggravating or relieving factors, no change in bowel habit, melena, weight loss, or lower urinary tract symptoms.

He doesn't drink, smoke, or use drugs in any way. His birth history, on the other hand, could not be examined further.

A physical examination revealed a patient who appeared to be in good health, with a blood pressure of 130/80 mm Hg, a heart rate of 75 beats per minute (bpm), and a temperature of 37.4°C. An abdominal examination revealed a suprapubic mass that was firm, non-tender, and immobile. His genitalia were examined and revealed the absence of the right testicle in the scrotum and in the inguinal canal, as well as a small testis in the left scrotum.

Paraclinical tests revealed no hematuria or proteinuria in the urine, a normal full blood count, no blast cells on the blood smear, and a negative serology for the human immunodeficiency virus (HIV) and hepatitis viral markers.

**Fig. 1:**
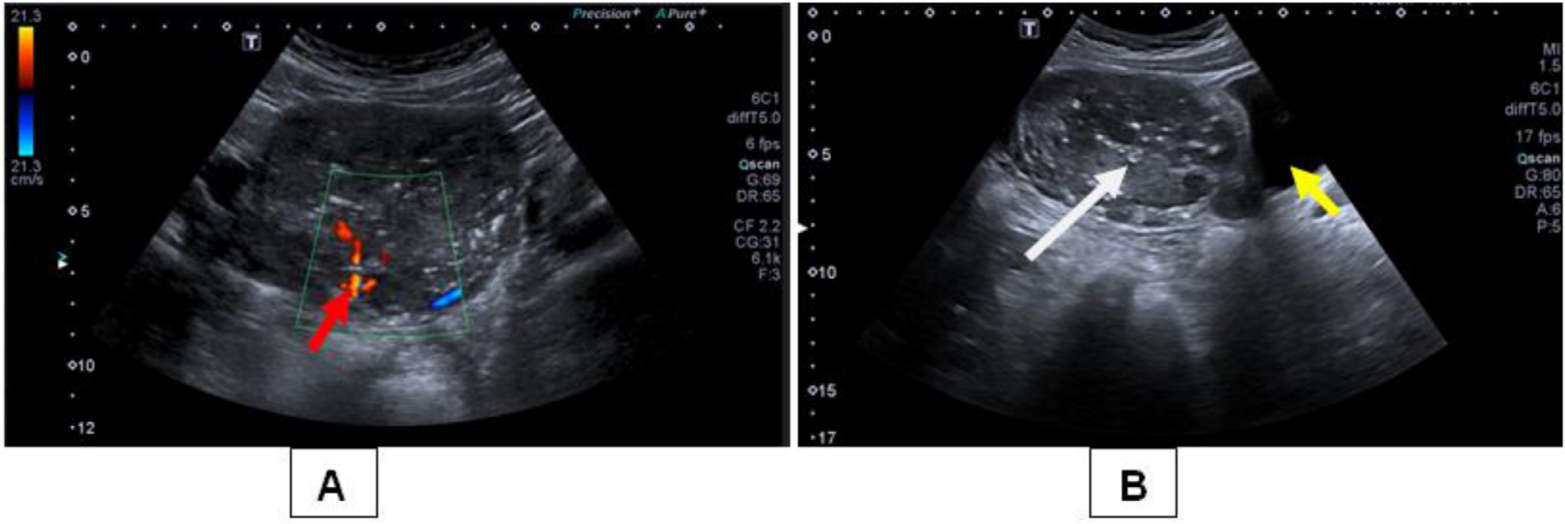
An ultrasound of the abdomen and pelvis revealed a 15-cm hypoechoic heterogeneous bean-shaped tumor (white arrow) above the bladder (yellow arrow) with internal vascularity (red arrow) and calcification.

**Fig. 2:**
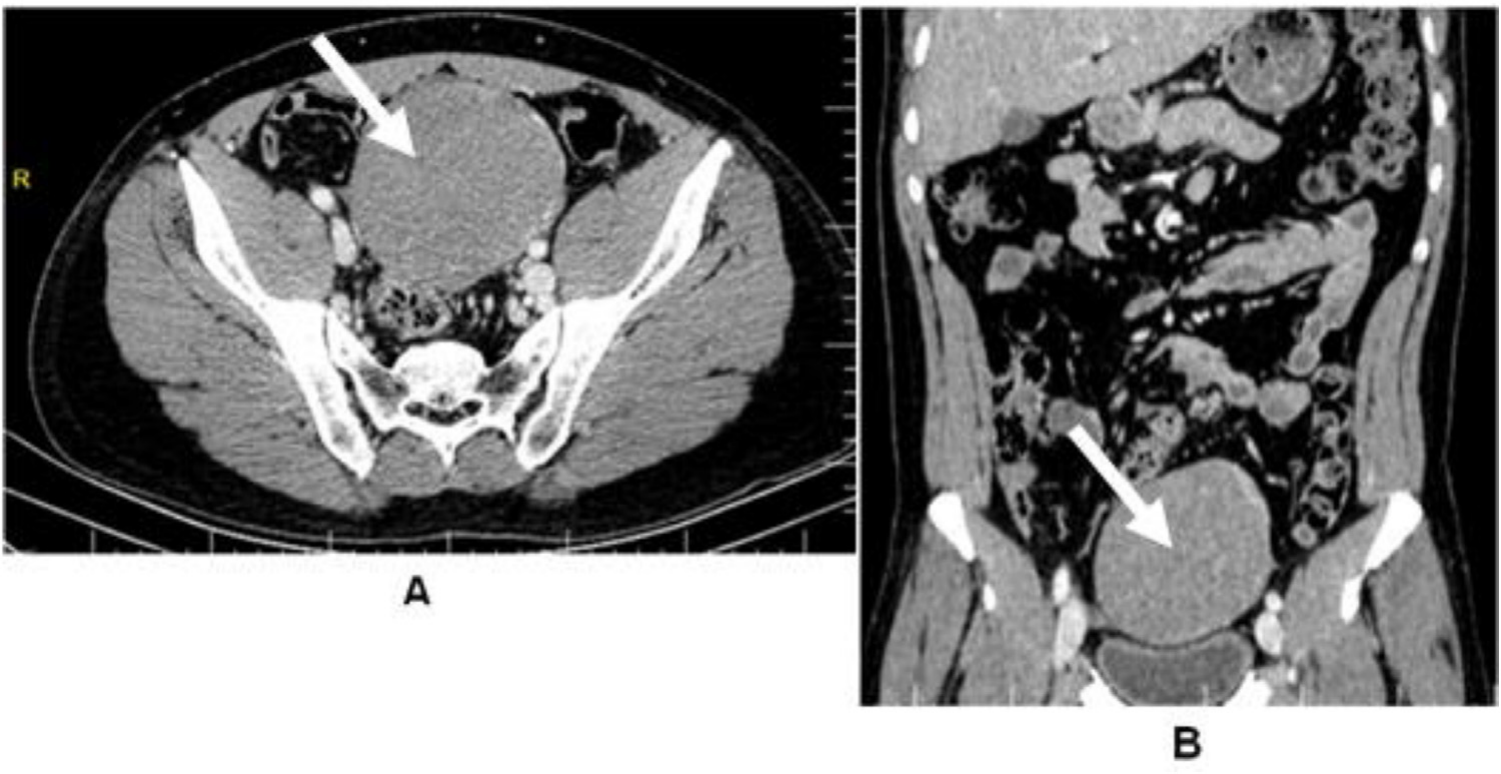
CECT abdomen pelvis axial images (A) and Coronal images (B) showed a well-defined round to oval approximately 10.1 cm × 9.7 cm × 8 cm heterogeneously enhancing mass (white arrow) in the pelvic region with vascular supply from the right testicular artery, indicating a seminoma of the undescended right testis.

An abdominopelvic ultrasound showed a 15-cm hypoechoic heterogeneous bean-shaped mass above the bladder with internal vascularity and calcification (Fig. 1). His liver, kidneys, spleen, pancreas, bladder, and bowels were all normal. Based on these findings, we suspected a desmoid tumor or an enlarged mesenteric lymph node However; the precise origin has yet to be determined. Contrast enhanced CT abdomen revealed a well-defined round to oval approximately 10.1 cm × 9.7 cm × 8 cm heterogeneously enhancing mass in the pelvic region with vascular supply from the right testicular artery, indicating a seminoma of the undescended right testis (Fig. 2). Plain X-rays and computed tomography of the chest did not reveal any distant metastatic lesions.

Following patient counseling and written consent, an open exploration was performed using a modified Gibson's incision. The right testicular mass was mobilized all around, vessels were ligated, and the specimen was removed.

In the histopathological examination, a seminoma of the undescended testis was discovered. On the postoperative fifth day, the patient was discharged.

Further management options were discussed with the patient, who was referred to an oncologist and andrologist for further evaluation.

## Discussion and conclusion

The testes grow in the abdomen throughout fatal development and then descend into the scrotal sac in the third trimester, usually between weeks 28 and 32. It may be stopped at any time along its path (cryptorchidism) or migrate to an abnormal side throughout the descent (ectopic testis).

Because of the rapid increase in tumor size and free mobility, as well as androgen function deficiencies. Such an organ is at high risk of torsion, trauma, infertility, and malignancy [4,6].

Malignant intraabdominal testes might present as anything from an asymptomatic, incidental mass to an acute abdomen caused by torsion or hemorrhage. The radiologic diagnosis is often challenging since the patient's history of cryptorchidism is not provided, and individuals who had cryptorchidism surgery as a kid are unaware that orchiectomy may not have been done [7].

Cryptorchidism should be treated at a young age, preferably prior to the first year, to prevent/early detect these types of tumors [2,3]. Early orchiopexy will preserve germinal epithelium, minimize infertility, and put the testis in a position that allows for easier self-examination and detection of testicular cancer.

In the case of an empty scrotum, an intra-abdominal mass should arouse concerns about an intra-abdominal testicular tumor. Adults with intra-abdominal testicular tumors are more likely to present at an advanced stage than those with intrascrotal testicular tumors. Cryptorchid testis should be investigated with CT or MRI to locate the testis and have orchiectomy performed if they are present in childhood. Such cases of intra-abdominal testicular cancer can only be prevented or detected early in this way.

## Ethics approval

Ethical approval was waived by the ethical committee of Mogadishu Somali Turkey, Recep Tayyip Erdogan Training and Research Hospital.

## Patient consent statement

Written informed consent was obtained from the patient for the publication of this case report and accompanying images.

## Availability of data and materials

The data that support the findings of this study are available in Mogadishu Somali Turkey, Recep Tayyip Erdogan Training and Research Hospital information system. Data are however allowed to the authors upon reasonable request and with permission of the education and research committee.
