# Erratum to “Intra-abdominal mass with empty scrotum in adult male revealed as testicular seminoma: A case report” [Radiology Case Reports 17 (2022) 3308–3311]

**DOI:** 10.1016/j.radcr.2023.12.038

**Published:** 2024-01-12

**Authors:** Yahye Garad Mohamed, Najib Mohamed Salad, Abdinasir Mohamed Elmi, Abdihamid Mohamed Ali

**Affiliations:** a Radiology Department, Mogadishu Somali Turkey, Training and Research Hospital, Mogadishu, Somalia; b General Surgery Department, Mogadishu Somali Turkey, Training and Research Hospital, Mogadishu, Somalia

The Publisher would like to inform you that during the publication process the declaration of competing interests was reproduced incorrectly. The correct statement is below.

**Competing Interests:**

The authors have no affiliation with any organization with a direct or indirect financial interest in the subject matter discussed in the manuscript.

The Publisher apologizes for any inconvenience caused by this mistake.

